# Residual fMRI sensitivity for identity changes in acquired prosopagnosia

**DOI:** 10.3389/fpsyg.2013.00756

**Published:** 2013-10-18

**Authors:** Christopher J. Fox, Giuseppe Iaria, Bradley C. Duchaine, Jason J. S. Barton

**Affiliations:** ^1^Departments of Medicine (Neurology) and Ophthalmology and Visual Sciences, University of British ColumbiaVancouver, BC, Canada; ^2^Departments of Psychology and Clinical Neurosciences, Hotchkiss Brain Institute, University of CalgaryCalgary, AB, Canada; ^3^Department of Psychological and Brain Sciences, Dartmouth CollegeHanover, NH, USA

**Keywords:** face perception, identity, expression, fMRI, adaptation, sensitivity, prosopagnosia

## Abstract

While a network of cortical regions contribute to face processing, the lesions in acquired prosopagnosia are highly variable, and likely result in different combinations of spared and affected regions of this network. To assess the residual functional sensitivities of spared regions in prosopagnosia, we designed a rapid event-related functional magnetic resonance imaging (fMRI) experiment that included pairs of faces with same or different identities and same or different expressions. By measuring the release from adaptation to these facial changes we determined the residual sensitivity of face-selective regions-of-interest. We tested three patients with acquired prosopagnosia, and all three of these patients demonstrated residual sensitivity for facial identity changes in surviving fusiform and occipital face areas of either the right or left hemisphere, but not in the right posterior superior temporal sulcus. The patients also showed some residual capabilities for facial discrimination with normal performance on the Benton Facial Recognition Test, but impaired performance on more complex tasks of facial discrimination. We conclude that fMRI can demonstrate residual processing of facial identity in acquired prosopagnosia, that this adaptation can occur in the same structures that show similar processing in healthy subjects, and further, that this adaptation may be related to behavioral indices of face perception.

## Introduction

Prosopagnosia is a neurological syndrome characterized by the failure to recognize familiar faces in the absence of more pervasive dysfunction of vision or memory (Barton, 2003). Patients with the acquired form can have a variety of lesions, most often damage to inferomedial occipitotemporal cortex, either bilaterally or in the right hemisphere only (Bodamer, 1947; Landis et al., 1986; Barton, 2003). Functional magnetic resonance imaging (fMRI) studies have shown a number of face-selective regions in the occipital and temporal lobes (Kanwisher et al., 1997; Haxby et al., 2000; Ishai et al., 2005), including the fusiform face area (FFA), the occipital face area (OFA), and the posterior superior temporal sulcus (pSTS) in both right and left hemispheres (Haxby et al., 2000). These regions are proposed by some as an anatomic “core” for face processing (Gobbini and Haxby, 2007). It seems probable that damage to these regions is involved in at least some if not most cases of acquired prosopagnosia, but the extent of damage to the various modules of this network in prosopagnosia is not yet known. Given the variety of lesions associated with prosopagnosia (Barton, 2008a,b), it is also likely that patients will differ in both modules affected and modules spared (de Gelder et al., 2003; Rossion et al., 2003a,b).

One question of interest is the residual function of spared regions of the face network in prosopagnosia. Identifying surviving face-selective regions in acquired prosopagnosia with a standard contrast between viewing faces and viewing objects (Rossion et al., 2003a,b) does not tell us the type of face information being processed by spared regions. Faces are a source of many types of information, including identity, expression, gaze direction, attractiveness, age and gender, among others. Cognitive models often segregate these different types of information into separate processing streams (Bruce and Young, 1986). Current anatomic models go even further and attempt to link specific functions to specific regions, for example, initial perception of facial structure in the OFA, perception of facial identity in the FFA, and perception of facial expression in the pSTS (Haxby et al., 2000). However, this segregation of function may not be as complete as the model suggests: a number of studies have shown some sensitivity to facial identity in the OFA (Rossion et al., 2003a,b; Avidan et al., 2005) and the pSTS (Winston et al., 2004; Fox et al., 2009a,b) on the one hand, and to facial expression in the FFA (Vuilleumier et al., 2004; Ganel et al., 2005; Fox et al., 2009a,b) on the other. In prosopagnosia, where patients have lost the ability to recognize facial identity, one can ask (1) which, if any, surviving face-selective modules still show sensitivity to identity, and (2) whether this correlates with residual ability to discriminate facial identity on behavioral tests.

One method used to assess the specific function of cortical regions is fMRI adaptation (Grill-Spector et al., 2006). This technique has shown that the fMRI BOLD signal declines with repeated presentations of identical stimuli. Furthermore, the technique can be exploited to determine what aspects of a stimulus are being processed in a region, by varying one stimulus property or dimension while keeping others constant. If the repeated stimuli vary only along a dimension that is irrelevant to the processing performed by a specific region, adaptation will still occur. However, if the varying dimension is being processed in this region, then repeated presentations will be treated as different stimuli, and no adaptation will be found (i.e., a “release from adaptation” will occur). In this way it is possible to determine what aspect of a stimulus is of interest to a cortical region. This method has been used in healthy subjects to demonstrate sensitivity to structural changes in a face within the OFA (Rotshtein et al., 2005), sensitivity to identity changes in the FFA (Winston et al., 2004; Rotshtein et al., 2005), and sensitivity to expression changes in the pSTS (Winston et al., 2004).

To date, there has been only one study of fMRI adaptation in an acquired prosopagnosic patient, patient PS. This study found residual sensitivity to facial identity changes, not in the spared right FFA, but in an object-selective region of the ventral lateral occipital cortex (Schiltz et al., 2006; Dricot et al., 2008). A similar fMRI adaptation study in four congenital prosopagnosic subjects found sensitivity to facial identity in both the undamaged OFA and FFA (Avidan et al., 2005). In contrast, a case of congenital “prosopamnesia” showed normal adaptation to familiar faces but not to unfamiliar faces in the right FFA (Williams et al., 2007).

Of note, the adaptation effects seen in the congenital prosopagnosia study were reported for the group, not for each subject (Avidan et al., 2005). While it may be valid to group congenital prosopagnosic subjects who have no apparent neurological lesion, the heterogeneity of damage in acquired prosopagnosia (Barton, 2003) makes group analyses difficult to interpret. Thus, it is important to design an fMRI adaptation method that can reveal significant sensitivity to identity or expression changes in an individual. The power of group analyses lies in the averaging of results across a number of subjects (Friston et al., 1999). In a similar fashion, averaging across multiple scans within a single subject can increase the power to detect a significant effect in that subject. By performing and averaging across multiple adaptation scans in each individual, we aimed to identify significant adaptation effects in single subjects.

Our goal was to use such a method to determine whether surviving face-selective regions of individuals with acquired prosopagnosia had any residual sensitivity to facial identity and/or expression. We assessed three patients on a wide array of behavioral tests to characterize their face processing deficits, and in particular their residual behavioral sensitivity to facial structure. All three patients then underwent fMRI testing, first with a face-localizer to determine which regions of the core face network (bilateral OFA, FFA, and pSTS) had or had not survived their lesion, and then with our adaptation paradigm to determine the residual sensitivity to identity and expression changes in these surviving regions. Given current models, we hypothesized that we would find residual sensitivity for identity changes in the right FFA, and for expression changes in the right pSTS. In addition, we hypothesized that residual sensitivity in the fMRI experiment may be indicative of a residual ability of prosopagnosic subjects to discriminate the structural properties of faces, as determined by our own experimental tests and standard neuropsychological instruments such as the Benton Face Recognition Test (Benton and van Allen, 1972).

## Methods

### Patients

Three brain-damaged patients with acquired prosopagnosia participated in this study. Informed consent was obtained and the protocol was approved by the institutional review boards of the University of British Columbia and Vancouver General Hospital, in accordance with The Code of Ethics of the World Medical Association, Declaration of Helsinki (Rickham, 1964). The focus of this research was to demonstrate the presence of residual sensitivity within face-selective regions of cortex in prosopagnosic individuals using an adaptation paradigm. Our goal was not to compare this residual sensitivity to the general population but rather simply to determine whether or not we could definitively demonstrate the presence of such a phenomenon in these brain-damaged individuals. [For data from three healthy right handed control subjects (C01-28 year old male, C02-34 year old male, C03-27 year old female) with normal or corrected-to-normal vision and no history of neurological disorders please see Supplemental Figure 1].

All patients had detailed neuropsychological and neurological examinations, supplemented with Goldmann perimetry and Farnsworth-Munsell 100-hue tests. The tests used to characterize their face perceptual abilities are listed in Table 1. Face perception is commonly segmented into a number of different cognitive processes, ranging from the early processing of facial structure relevant to the perception of (1) facial identity or (2) facial expression, to latter stages of facial memory which can be accessed both (3) overtly and (4) covertly. First, identity perception was assessed with the Benton Facial Recognition Test (Benton and van Allen, 1972) and with a 3-alternative forced-choice oddity test (chance = 33%) for discriminating identity changes in morphed facial stimuli (Fox et al., 2011). Importantly, normal scores on the Benton Facial Recognition Test do not necessarily indicate normal identity perception (Farah, 1990; Duchaine and Weidenfeld, 2003), and therefore, more weight should be given to performance on the morphed-face discrimination test, which has been shown to be a more sensitive measure of impaired perceptual processing (Fox et al., 2011). Second, expression perception was assessed with the revised version of the Reading the Mind in the Eyes Test (Baron-Cohen et al., 2001), and with a forced-choice oddity test of the discrimination of morphed-expression changes, equivalent in difficulty to the oddity test for morphed-identity changes (Fox et al., 2011). Third, overt short-term facial memory was assessed with the Warrington Recognition Memory Test (Warrington, 1984), and long-term facial memory with a Famous Face Recognition Test that required subjects to indicate which of a series of 20 famous and 20 anonymous faces was familiar (Barton et al., 2001). This test included a similar series of 20 famous and 20 unfamiliar names with the patient selecting the famous name and then providing semantic information about the name to ensure that semantic memory stores were intact. A 37-item facial imagery test was also used to assess the adequacy of facial memory stores independent of the status of perceptual processes (Barton and Cherkasova, 2003). Fourth, covert facial memory was assessed with two tests using a direct strategy, a name-cued forced-choice test that showed subjects a famous face (that they claimed not to recognize) paired with an anonymous one and asked them to indicate which was the face named by the examiner, and an indirect strategy, an occupation-sorting test that required subjects to sort famous faces they did not recognize on the basis of whether they were politicians or actors (Barton et al., 2001).

**Table 1:**
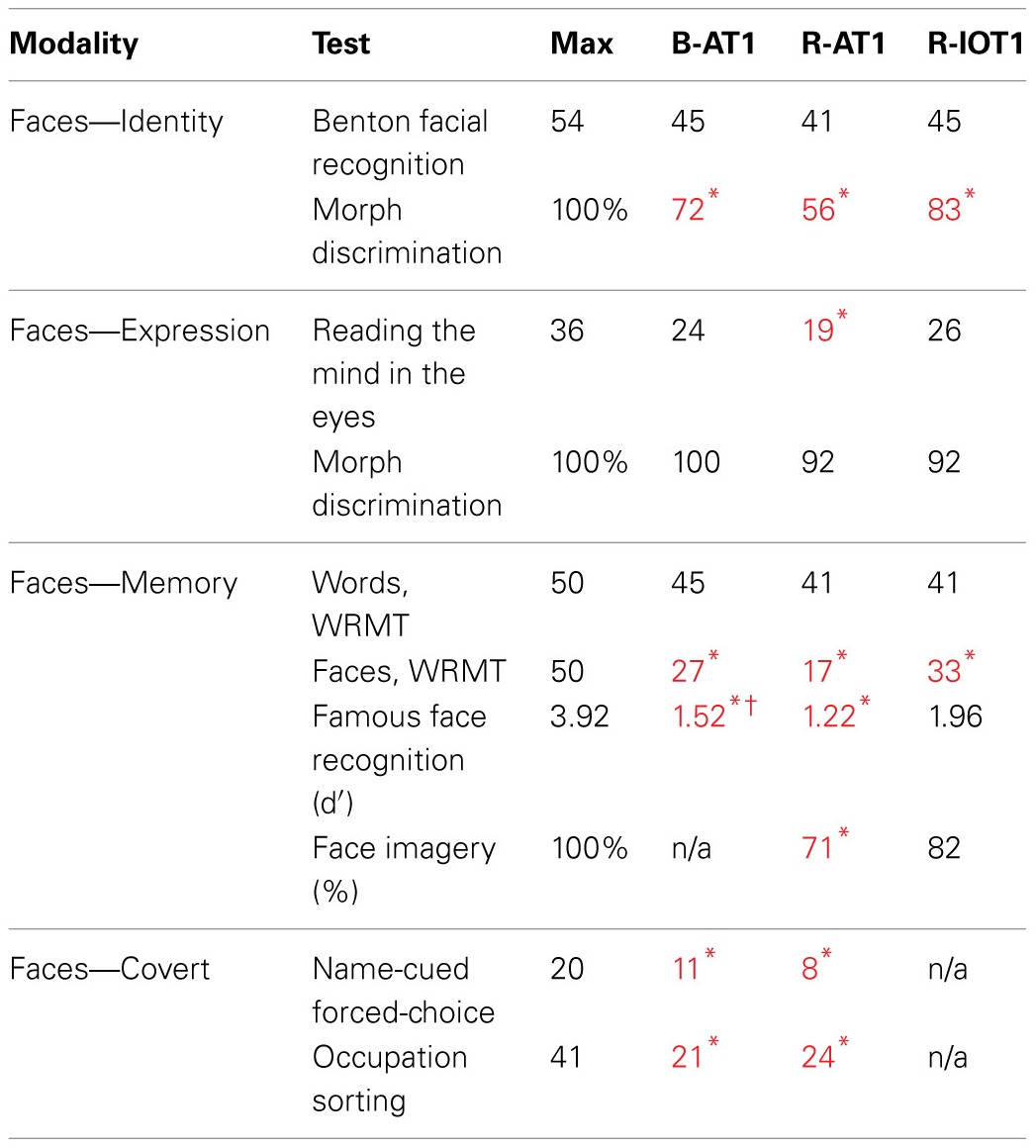
**Results from the battery of face tests**.

Impairments are indicated in red.

(WRMT = Warrington Recognition Memory Test). For normative data on these previously published tests please consult the appropriate references included herein.

^†^Due to poor knowledge of celebrities, a version of this test using personally familiar faces was given to B-AT1.

**Figure 1:**
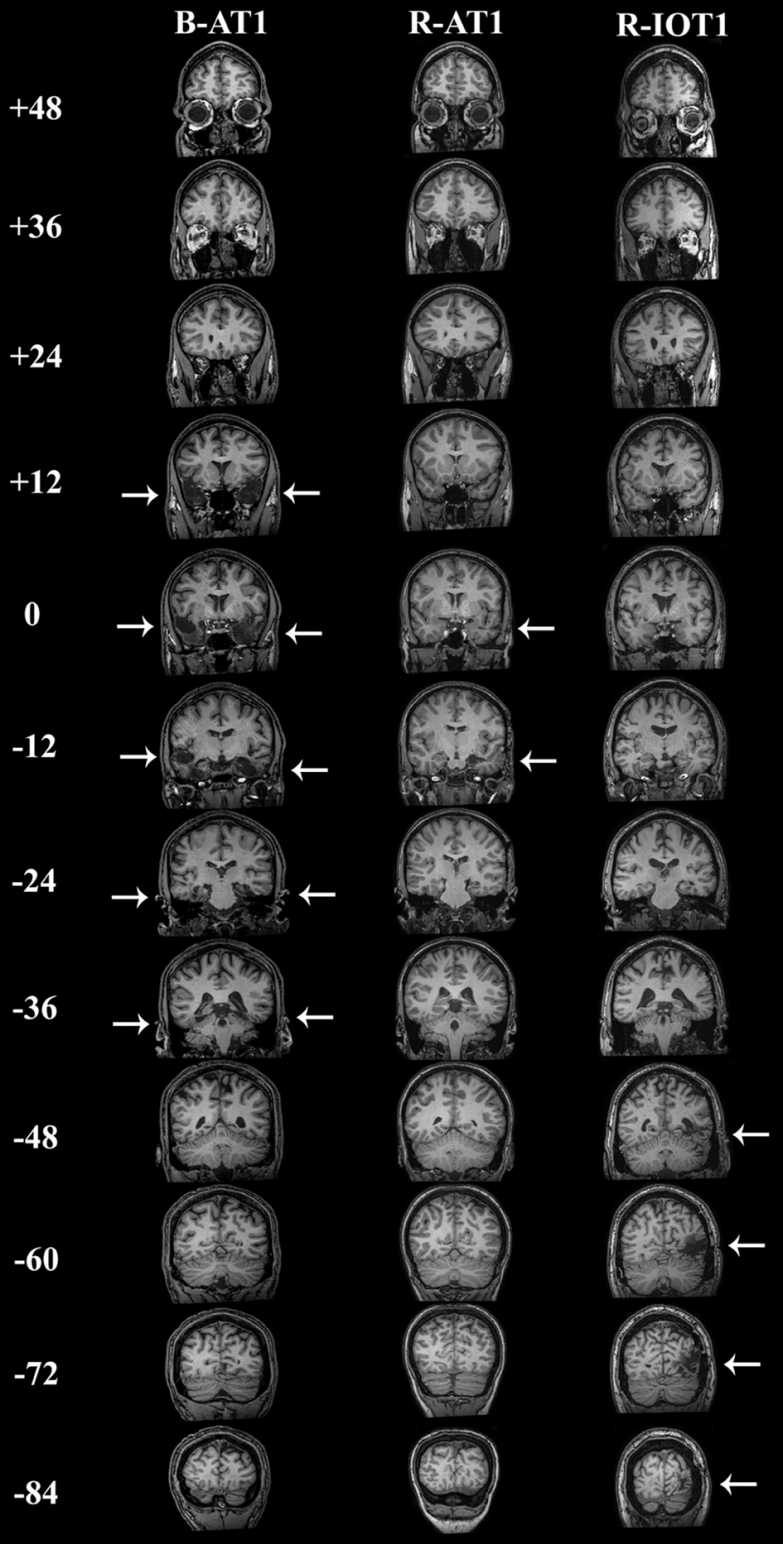
**Coronal T1-weighted MRI brain images of the three patients, standardized to Talairach space**. Slices were taken every 12 mm, from *y* = + 48 mm to *y* = −84 mm. B-AT1 has large bilateral lesions of the anterior temporal lobes following herpes encephalitis (+12 to −36 mm). R-AT1 has a small surgical lesion in the right anterior temporal lobe, additionally affecting the right hippocampus and amygdala (0–12 mm). R-IOT1 has a single right inferior occipitotemporal lesion from his prior hemorrhage (−48 to −84 m).

The first patient, identified as B-AT1 (B = bilateral; AT = anterior temporal,) is a 24 year-old right-handed male who had herpes simplex encephalitis three years prior (Figure 1). Since recovery, he has noted extreme difficulty in recognizing and learning faces, though he can recognize some family members. General memory and mental functioning is unaffected, allowing him to attend college and hold full-time employment. He has mild topographagnosia, and mild anomia for low-frequency items (although semantic knowledge of these items is evident). He had acuity of 20/20 and normal visual fields. He performed normally on the Benton Facial Recognition Test, but was mildly impaired in discrimination of morphed-identity changes. Facial expression processing was unaffected. He was severely impaired on the Faces component of the Warrington Recognition Memory Test, but not the Words component. He did poorly on a modified familiar face recognition test that used pictures of his relatives rather than celebrities, due to limited knowledge of the latter (which also invalidated the test of facial imagery). He showed no evidence of covert recognition on either the name-cued forced-choice or the occupation-sorting test.

The second patient, R-AT1 (R = right hemisphere; AT = anterior temporal), is a 24 year-old right-handed female. One year prior to testing she had a selective right amygdalohippocampectomy for epilepsy (Figure 1), following which she has had difficulty recognizing faces, needing to rely on voice or other means to recognize individuals. General mental functioning was intact: she is currently attending university, although she has problems with visual memory and relies on verbal strategies to study. She had acuity of 20/20 and normal visual fields. She performed normally on the Benton Facial Recognition Test (Table 1), but was impaired on the more difficult discriminations of morphed-identity changes. The Reading the Mind in the Eyes Test suggested reduced recognition of expression, but the perception of morphed-expression changes was normal. She was impaired on the Faces but not the Words component of the Warrington Recognition Memory Test. Face recognition was reduced on the Famous Face Recognition Test and she had reduced facial imagery. There was no evidence for covert face recognition on either the name-cued forced-choice or the occupation-sorting tests.

The third patient, R-IOT1 (R = right hemisphere, IOT = inferior occipitotemporal), is a 49 year-old left-handed male who twelve years prior had suffered an occipital cerebral hemorrhage from rupture of an arteriovenous malformation (Figure 1). Immediately following this event he complained of trouble recognizing hospital workers and needed to rely on hairstyle, facial hair, or voice for person recognition, a problem that has not resolved. He also displayed letter-by-letter reading immediately after the hemorrhage but this had resolved quickly. On examination his acuity was 20/20 and he had a left superior quadrantanopia and mild topographagnosia. He performed normally on most face tests, including the Benton Face Recognition Test (Table 1), but was mildly impaired on the discrimination of morphed-identity changes. He did better on the Famous Face Recognition Test than any other prosopagnosic patient, but claimed that because we used well-known images, he was recognizing the pictures and not the people (because he recognized these images, he also could not do the covert tests, as they used similar images). In support of this, he was significantly impaired on a famous faces test using less typical images of celebrities [11/25; (Duchaine, 2000)] and on the Faces (but not the Word) component of the Warrington Recognition Memory Test, which tests short-term recognition with anonymous people. Facial expression processing was unaffected.

### Stimuli

**Figure 2:**
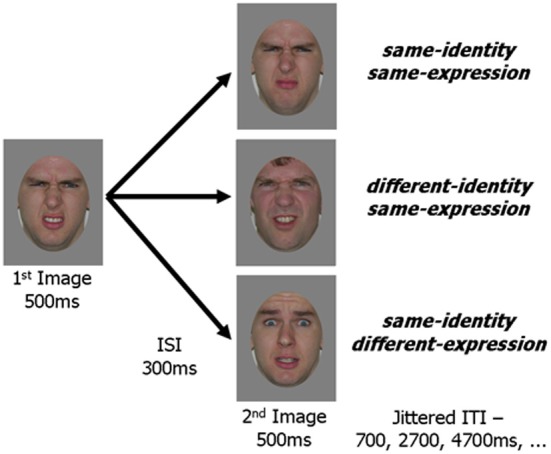
**Schematic representation of an experimental trial**. In all three experimental conditions the first image was the same. The second image in the pair was either a new picture with the same identity and same expression as the first image, a picture of a different person with the same expression or a picture of the same person with a different expression. An image pair was presented within every *TR* (2 s) and fixation trials were randomly intermixed with experimental trials.

Face images were selected from the Karolinska Database of Emotional Faces (Lundqvist and Litton, 1998) and from our laboratory's collection. All images were cropped about the face and uniformly sized to 512 by 634 pixels. A standard gray oval was placed over each face to occlude the neck, hairline and picture background while leaving internal facial features and external face contour unaffected (Figure 2). Quartets of face images were selected such that for a given image, a second image showed the same identity with a different version of the same expression, a third image showed the same identity with a different expression, and a fourth image showed a different identity (of the same gender as the first image) displaying the same expression as the given image. Forty such quartets were created, 20 using female faces and 20 using male faces. Five facial expressions were included amongst the faces (anger, fear, happiness, sadness, disgust) with each expression appearing ten times (5 for each gender) as the base expression (displayed in 3 of the 4 images) and 10 times as the different expression (displayed in 1 of the 4 images).

### Design

Images from each of the 40 face quartets were paired to create the three experimental conditions. The same image was always presented as the first in each pair with the second image varying between conditions: *same-identity/same-expression, different-identity/same-expression, same-identity/different-expression*. This resulted in 40 unique trials for each of the three experimental conditions.

Six other faces (3 males, 3 females), which were different from the faces used in the experimental conditions, displaying 3 different expressions (anger, fear, happiness) were selected and formatted in a gray oval as described above. Upright and inverted versions of these six faces were created. Two face pairs were formed from each of the six identities; upright-inverted and inverted-upright. These 12 pairs became target trials in the fMRI adaptation experiment.

### Procedure

An experimental trial consisted of a pair of faces presented within each repetition time (*TR* = 2 s). The first face was presented for 500 ms and followed by a 300 ms inter-stimulus-interval (ISI). This was followed by a 500 ms presentation of the second face and a 700 ms inter-trial-interval (ITI). In order to avoid retinal adaptation image location randomly varied from image to image within a region of 50 by 50 pixels.

For each experimental scan 32 of the 40 face quartets were randomly selected, and all 3 experimental trials (one from each condition: *same-identity/same-expression, different-identity/same-expression, same-identity/different-expression*) from these quartets were presented during the scan. This resulted in 32 experimental trials per condition (from the 32 randomly selected face quartets) and 96 trials total. In addition to these experimental trials 10 of the 12 target trials (i.e., inverted faces) were randomly selected and included. Participants were asked to respond to the inverted face in these target trials with a keypress, which acted as a means to ensure subjects attended to the faces. Finally, 48 fixation trials, in which the face images were replaced by a fixation cross, were randomly interspersed among the experimental and target trials, producing the jittering required for rapid event-related experimental designs (Grill-Spector et al., 2004; Serences, 2004). The same procedure of random selection and randomized trial order was used to create six different experimental scans. Each experimental scan began with 1 fixation trial and ended with 6 fixation trials. All six experimental scans were presented to each participant in random order.

### fMRI

Structural and functional MRIs were performed on all participants. All scans were acquired in a 3.0 Tesla Philips scanner. Stimuli were presented using Presentation 9.81 software and were rear-projected onto a mirror mounted on the head coil. Whole brain anatomical scans were acquired using a T1-weighted echoplanar imaging (EPI) sequence, consisting of 170 axial slices of 1 mm thickness (1 mm gap) with an in-plane resolution of 1 mm × 1 mm (FOV = 256). T2-weighted functional scans (*TR* = 2 s; *TE* = 30 ms) were acquired using an interleaved ascending EPI sequence, consisting of 36 axial slices of 3 mm thickness (1 mm gap) with an in-plane resolution of 1.875 mm × 1.875 mm (FOV = 240).

We used a dynamic localizer that presented videos of moving faces and moving objects (Fox et al., 2009a,b) to identify regions of the core face network (i.e., right and left OFA, FFA, and pSTS) (Haxby et al., 2000). This localizer contrasts video-clips of faces changing in expression (i.e., from neutral to happy) with those of objects undergoing types of motion without large translations in position (i.e., basketball rotating). Video-clips of objects were gathered from the internet, and video-clips of faces were provided by Chris Benton, Department of Experimental Psychology, University of Bristol, UK (Benton et al., 2007), with all video-clips resized to a width of 400 pixels. Prior work in our laboratory demonstrated that this dynamic localizer is more sensitive in localizing regions of the core face network (98% success rate) than the standard technique which contrasts static images of faces and objects (Fox et al., 2009a,b). Importantly work from other laboratories also suggests that a dynamic signal can act to enhance facial identity recognition in prosopagnosic patients (Longmore and Tree, 2013) making dynamic stimuli a more appropriate choice to activate the core face network. Patients performed a “one-back task”: that is, they pressed a button if a video was identical to the previous one. Fixation blocks began and ended the session and were alternated with image blocks, with all blocks lasting 12 s. Eight blocks of each image category (object, face) were presented in a counterbalanced order. Each image block consisted of 6 video-clips (5 novel and 1 repeated) presented centrally for 2000 ms each. The dynamic localizer was followed by presentation of the six experimental scans.

The first volume of each functional scan was discarded to allow for scanner equilibration. All MRI data were analyzed using BrainVoyager QX Version 1.8 (www.brainvoyager.com). Anatomical scans were not preprocessed, but were standardized to Talairach space (Talairach and Tournoux, 1988). Preprocessing of functional scans consisted of corrections for slice scan time acquisition, head motion (trilinear interpolation), and temporal filtering with a high pass filter in order to remove frequencies less than 3 cycles/time course. Functional scans were individually co-registered to their respective anatomical scan, using the first retained functional volume to generate the co-registration matrix.

The dynamic localizer time course was analyzed with a single subject GLM, with objects (O) and faces (F) as predictors, and a F > O contrast was overlaid on the whole brain. Using a False-Discovery-Rate of *q* < 0.05 (corrected for multiple comparisons), we identified the core regions of face perception, bilaterally, within each participant (Haxby et al., 2000). Contiguous clusters of face-selective voxels located on the lateral temporal portion of the fusiform gyrus were designated as the FFA, while clusters located on the lateral surface of the inferior occipital gyrus were designated as the OFA. Face-selective clusters located on the posterior segment of the superior temporal sulcus were designated as the pSTS. Following a technique to maximize face-selectivity in each region-of-interest (ROI) (Fox et al., 2009a,b), we selected the 50 voxels, contiguous with the peak voxel, that displayed the highest *t*-value for the F > O contrast. These 50 voxel clusters were then subject to the experimental analyses.

Experimental MRI scans were analyzed using a deconvolution analysis that accounts for non-linear summation of the blood oxygen level dependent (BOLD) response in rapid event-related designs. The deconvolution analysis samples BOLD activity at trial onset (time = 0 s) and a further 9 times in 2 s intervals, resulting in an unbiased model of the hemodynamic response (HDR). The inverted target trials were included as a separate condition in the deconvolution analysis, to account for all non-fixation trials, but were not included in subsequent analyses.

**Figure 3:**
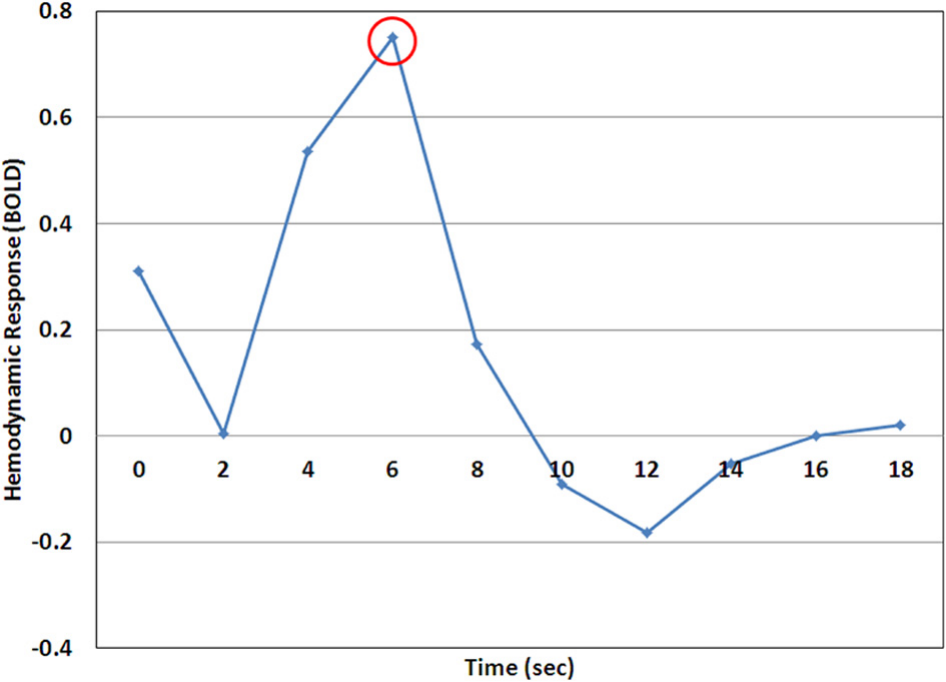
**A representative example of the hemodynamic response (HDR) as calculated by the deconvolution analysis**. In this case the time point at 6 s (encircled) would be considered the HDR-peak and the value at this time point would be used for analysis.

Within each ROI, results from the six experimental scans were combined using a multi-study GLM function that used the three experimental conditions (*same-identity/same-expression, different-identity/same-expression*, and *same-identity/different-expression*) as functions within the GLM (BrainVoyager). While one cannot determine the significance of differences in a single scan in a single subject, averaging across multiple scans enables the assessment of statistical significance in the single subject. Significant adaptation of the HDR may take a number of forms including a reduced HDR-peak due to neural fatigue or a narrowing of the full-HDR due to a facilitated neural response (Grill-Spector et al., 2006). To examine both possibilities we first collapsed data across all three experimental conditions. Then, within each ROI, the full-HDR was defined as the sum of all consecutive time points that showed a significant increase from baseline (*p* < 0.05, 1-tailed). The HDR-peak was defined as the time point exhibiting a maximal increase in BOLD activity, or the average of this time point and adjacent time points that did not significantly differ (*p* > 0.05, 1-tailed). Using these definitions, the values of the full-HDR and HDR-peak were then determined for each of the three experimental conditions. Contrasts of the *different-identity/same-expression > same-identity/same-expression* and the *same-identity/different-expression > same-identity/same-expression* were performed, using the multi-study GLM, to assess identity and expression adaptation, respectively. Significant release from adaptation in the *different* conditions was set at α < 0.05, and would indicate sensitivity of the ROI to changes in identity or expression. Only positive release from adaptation values indicate sensitivity to the varied stimulus; negative values would suggest priming of an ROI to the presented stimulus and are not discussed herein (in fact only one control demonstrated a negative release from adaptation in the L-FFA). The difference values resulting from these two contrasts are presented graphically. As all effects in the full-HDR condition were replicated in the HDR-peak condition, but were stronger in the latter, we only present the results of the HDR-peak analyses (Figure 3). Release from adaptation is therefore, defined as a difference in peak beta values from the modeled HDR, with the specific contrasted conditions outlined above.

## Results

**Figure 4:**
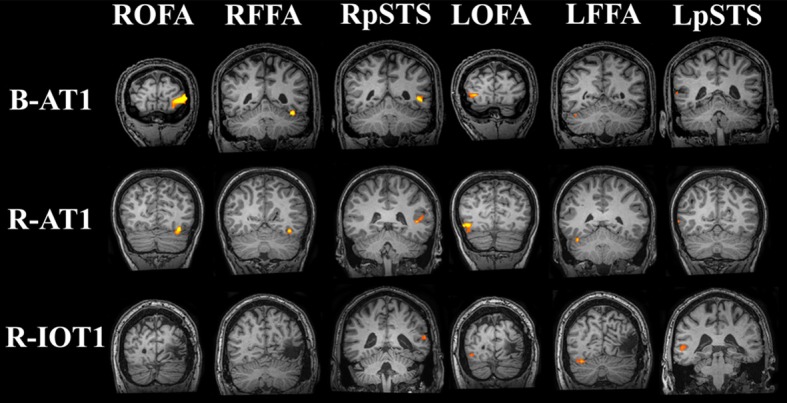
**Core system regions-of-interest identified with the functional localizers (all brains standardized to Talairach space)**. All six regions of the core system were identified in B-AT1 and R-AT1. Due to the location of the lesion, R-IOT1 does not display a right OFA or right FFA. However, a right posterior STS (pSTS) was identified along with all three core regions in the left hemisphere.

**Figure 5:**
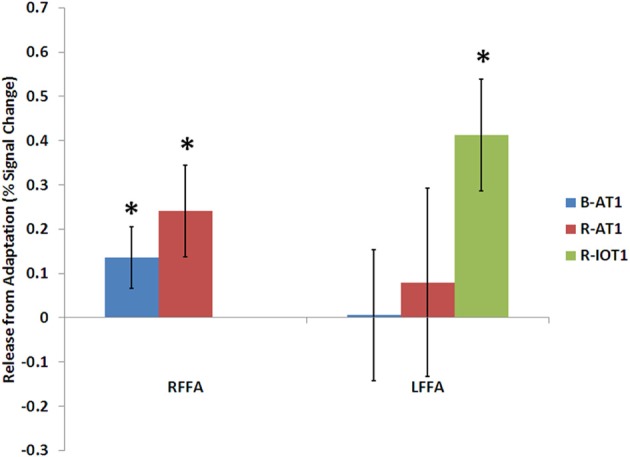
**Release from adaptation in response to identity changes as seen in the fusiform face areas of the three patients**. Significant release from adaptation was observed in the right FFA of both B-AT1 and R-AT1. In contrast, R-IOT1 who did not have a right FFA due to damage, exhibited significant release from adaptation in the left FFA. ^*^p < 0.05.

**Figure 6:**
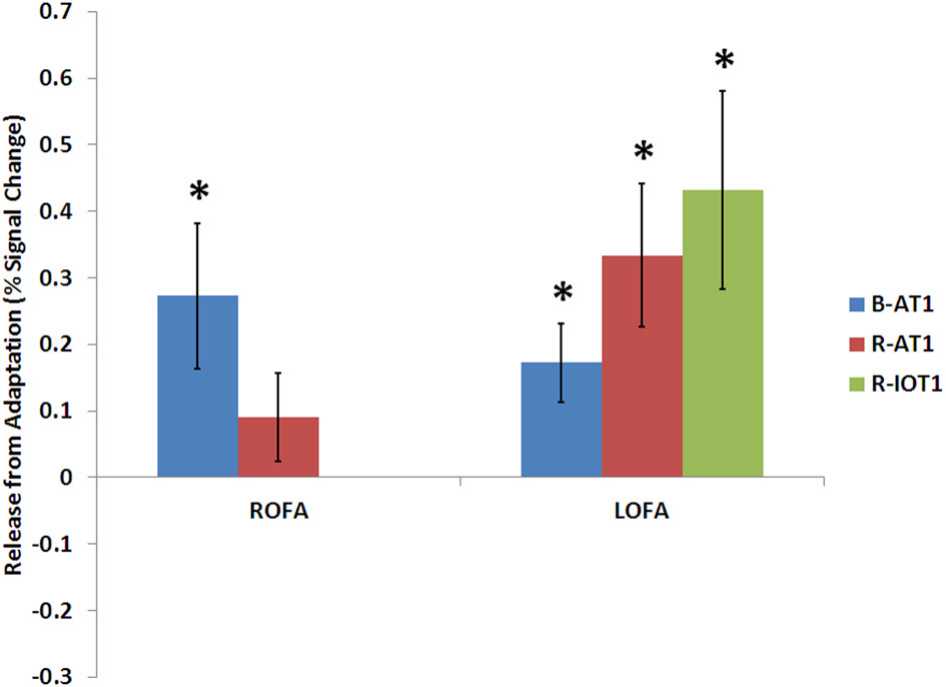
**Release from adaptation in response to identity changes as seen in the occipital face areas of the three patients**. Significant release from adaptation was observed in the left FFA of all three patients and only in the right OFA of B-AT1 (R-IOT1 did not have a right OFA due to damage). ^*^p < 0.05.

B-AT1 has extensive bilateral damage to the anterior temporal lobes, which extends to the inferior surface of the middle temporal lobe (Figure 1). Functional MRI located all six regions of the core face-processing system (Table 2; Figure 4). Release from adaptation when identity changed was found in the right FFA (0.14 ± 0.07, *p* < 0.05; Figure 5) and in the right (0.27 ± 0.11, *p* < 0.05) and left (0.18 ± 0.06, *p* < 0.005) OFA (Figure 6). No sensitivity to expression changes was observed.

**Table 2:** **Results of the dynamic functional localizer, with brains standardized to Talairach space**.

**Subject**	**Region**	**Maximum**	**Minimum**	***X***	***Y***	***Z***
		***t*-value**	***t*-value**			
B-AT1	**ROFA**	**12.37**	**11.18**	**30**	**−88**	**−5**
	**RFFA**	**13.09**	**10.25**	**39**	**−52**	**−20**
	**RpSTS**	**9.67**	**7.62**	**45**	**−49**	**−2**
	LOFA	9.43	7.45	−30	−85	−8
	LFFA	5.96	5.04	−39	−55	−26
	LpSTS	5.9	4.95	−60	−46	4
R-AT1	**ROFA**	**14.88**	**11.27**	**27**	**−70**	**−20**
	**RFFA**	**11.29**	**6.46**	**36**	**−58**	**−11**
	**RpSTS**	**14.18**	**10.81**	**42**	**−40**	**4**
	LOFA	12.92	11.31	−42	−70	−8
	LFFA	11.90	9.99	−39	−43	−26
	LpSTS	11.66	8.81	−57	−46	13
R-IOT1	**ROFA**	**LESION**				
	**RFFA**	**LESION**				
	**RpSTS**	**5.52**	**3.67**	**57**	**−40**	**13**
	LOFA	6.50	4.85	−37	−82	−20
	LFFA	4.73	3.18	−33	−67	−23
	LpSTS	7.42	5.23	−42	−40	4

The peak 50 voxels were defined as the region-of-interest with maximum and minimum t-values reported. Right hemispheric regions of interest are bolded.

R-AT1 has a small lesion in the anterior right temporal lobe that affects the anterior hippocampus, amygdala, and overlying temporal cortex (Figure 1). All six ROIs of the core face processing system were identified (Table 2; Figure 4). Release from adaptation when identity changed was found in the right FFA (0.24 ± 0.10, *p* < 0.05; Figure 5) and the left OFA (0.33 ± 0.11, *p* < 0.005; Figure 6). No sensitivity to expression changes was observed.

R-IOT1 has a unilateral right lesion affecting both the occipital and posterior temporal cortex (Figure 1). The functional localizer failed to identify an OFA or FFA in the right hemisphere, though the right pSTS and all three regions in the left hemisphere were identified (Table 2; Figure 4). Release from adaptation when identity changed was observed in the left FFA (0.41 ± 0.13, *p* < 0.005; Figure 5), and the left OFA (0.43 ± 0.15, *p* < 0.005; Figure 6). No sensitivity to expression changes was observed.

## Discussion

### Residual sensitivity to identity changes in the fusiform face area

A surviving right FFA was found in two prosopagnosic patients (B-AT1 and R-AT1; Figure 4). In both it showed residual sensitivity to facial identity, with larger responses to different than to repeated identities (Figure 5). This sensitivity to identity is consistent with the role of the right FFA in identity processing in current models of face perception (Haxby et al., 2000), and prior fMRI adaptation studies using group-based analyses (Andrews and Ewbank, 2004; Winston et al., 2004; Rotshtein et al., 2005; Fox et al., 2009a,b). However, this finding contrasts with the only previous study of identity adaptation in acquired prosopagnosia (patient PS), which did not find such sensitivity in the spared right FFA (Schiltz et al., 2006; Dricot et al., 2008). An important difference is that both of our patients had damage limited to the anterior temporal lobes, with sparing of all six core regions of the face processing network, while PS had loss of the right OFA and left FFA (Rossion et al., 2003a,b). This suggests that residual sensitivity to face identity in the FFA may depend upon inputs from other surviving core face-processing regions, a hypothesis that should be tested in additional patients.

The left FFA did not show sensitivity to identity changes in either patient B-AT1 or R-AT1, but significant sensitivity was observed in the left FFA of R-IOT1, who differs from the others in that he is strongly left-handed (Figure 5). This raises the possibility of anomalous lateralization, as suggested in prior cases of prosopagnosia in left-handed individuals with unilateral left occipitotemporal lesions (Tzavaras et al., 1973; Mattson et al., 2000; Barton, 2008a,b). While all fMRI studies show smaller and less frequent face-selective activity in the left fusiform region than the right, it may be that the left FFA has a greater role than normal in face-processing in a left-handed subject like R-IOT1. If so, this could explain why adaptation effects for identity were found in the left FFA of R-IOT1 but not in the other patients.

### Residual sensitivity to identity changes in the occipital face area

Beyond the FFA, we also found identity adaptation in the OFA of our three patients (Figure 6). The right OFA is spared in B-AT1, and R-AT1 (Figure 4) but identity adaptation was found in the right OFA only for B-AT1 (Figure 6). In contrast, we observed identity adaptation in the surviving left OFA of all three patients (Figure 6). The OFA is traditionally thought to be involved in the early perception of facial structure prior to the decoding of facial identity (Haxby et al., 2000; Rotshtein et al., 2005; Fox et al., 2009a,b). While this ability to detect structural changes ultimately leads to identity recognition, it may be that the release from adaptation we observe in the OFA reflects response to a structural change at an early perceptual level and is not necessarily linked to a perceived identity change. However, while it is sometimes claimed that the OFA may encode facial structure relevant to both identity and expression, we did not find a similar release from adaptation when expression changed. In fact, none of the face-selective regions in any patient showed release from adaptation when expression changed, not even the right pSTS, which has shown such adaptation sensitivity to expression in previous group studies (Winston et al., 2004; Fox et al., 2009a,b). Failure to demonstrate adaptation for expression may have many origins, including lack of power in the individual subject, or even a requirement for enhanced attention, given that more pronounced activity is found for expression-based signals during expression-based tasks than during an irrelevant experimental task (Narumoto et al., 2001; Fox et al., 2009a,b). However, the fact that sensitivity to expression was not observed anywhere in this study leaves open the possibility that the sensitivity we report in the OFA is in fact a response to the structural differences between two different faces rather than sensitivity to the identity change itself, as the structural change between two identities is often more readily apparent than the structural change between two expressions. Importantly, adaptation effects for identity have not previously been reported or examined in the left OFA, thus, another possibility is that the sensitivity to identity changes we observe in the left OFA of these three patients may actually reflect a compensatory change in the face network of these brain-damaged patients much like the report which demonstrated identity adaptation effects in the ventral lateral occipital complex, a region not normally implicated in face processing, in the prosopagnosic patient PS (Dricot et al., 2008).

### No residual sensitivity in the posterior superior temporal sulcu

We did not find any identity adaptation in the right pSTS of any patient. Residual processing of identity in the STS has been suggested by some as a possible compensatory mechanism in prosopagnosia, particular by those who promote a dissociated dorsal route of face processing as an explanation for covert recognition (Tranel et al., 1995). While our behavioral tests did not show any covert face processing in any of these four patients, it should be stressed that the dissociable dorsal route has been advanced primarily by those studying autonomic indices of covert recognition (Bauer and Verfaellie, 1988; Tranel et al., 1995). Indeed, it may be that covert behavioral and covert autonomic measures index different phenomena, with the former emerging from residual function of the normal face-processing network, while residual electrodermal responsivity to faces may reflect activity in a separate pathway for mediating autonomic reactions to faces (Schweinberger and Burton, 2003). For these reasons, our data are limited in the conclusions that can be drawn regarding the anatomic correlates of covert face recognition. However, our data would at least suggest that following a variety of patterns of damage in prosopagnosia, residual sensitivity to face identity appears more likely in other components of the core face-processing network than in the pSTS.

Another possibility for the failure to identify adaptation to facial expression within the current design may be the restriction of our analysis to predefined ROIs. A recent study by Mur et al. (2010) demonstrated adaptation to repeated presentation of faces in areas outside the traditional face areas, including the parahippocampal place area and early visual cortex. They argue that this may represent an attentional affect rather than specific face-sensitivity within these regions. However, the possibility remains that the pSTS which we identified with our localizer did not in fact capture the collection of neurons that are most involved in expression recognition, and which would demonstrate a measurable release from adaptation with expression changes. Further experimentation with whole-brain analysis rather than predefined ROIs may identify just such a region.

### Residual sensitivity and behavioral performance

It is interesting to compare the patient's residual ability to discriminate faces of different identities and parallel these findings with the fMRI adaptation results for identity. B-AT1, R-AT1, and R-IOT1 all performed normally on the Benton Face Recognition Test and had mild to moderate deficits on the morph discrimination test for identity; on the fMRI experiment all showed identity adaptation effects in at least one face-selective region. In contrast, a prosopagnosic patient in another study, PS, was significantly impaired on the Benton Facial Recognition Test and showed no identity adaptation effects in the FFA (Rossion et al., 2003a,b). These results suggest that residual perceptual sensitivity to aspects of facial structure related to identity may have an anatomic correlate in the residual neural sensitivity of the FFA and OFA to these same structural properties.

In conclusion, we devised an fMRI adaptation protocol which can reveal significant adaptation to facial identity in the single subject. In three acquired prosopagnosics with a variety of lesions, we found residual sensitivity to identity in the spared right FFA of two right handed prosopagnosic patients with anterior temporal damage, and in the spared left FFA of one left-handed prosopagnosic patient who had loss of the right FFA and OFA. We also observed sensitivity to identity within the left OFA of these three patients, which may reflect either normal sensitivity to facial structure or a compensatory enhancement following damage to the face processing network. The presence of adaptation effects for identity paralleled residual ability to discriminate between different faces, as measured by the Benton Facial Recognition Test but not the more difficult morphed-face discrimination test. Further study in a larger cohort of subjects with either acquired or congenital prosopagnosia would be of interest.

### Conflict of interest statement

The authors declare that the research was conducted in the absence of any commercial or financial relationships that could be construed as a potential conflict of interest.

## Supplementary material

Supplemental Figure 1 **(A)** Control data for the *different-identity/same-expression > same-identity/same-expression* contrast. A significant release from adaptation (^*^) for identity changes was seen within the right FFA of C01 and C03, and within the left OFA of C03. A trend in the same direction (#) was observed in the right OFA of C03. **(B)** No significant release from adaptation was observed for changes in expression, following the *same-identity/different-expression > same-identity/same-expression* contrast. When compared to the data from the patient population we again see a release from adaptation to identity changes in the right FFA (2/3 controls) but there is no evidence of sensitivity to facial expression with this experimental design.
