# Supplementary figures and images for: Residual fMRI sensitivity for identity changes in acquired prosopagnosia
Source: Front Psychol. 2013 Oct 18;4:756. doi: 10.3389/fpsyg.2013.00756 (PMC3799008; doi:10.3389/fpsyg.2013.00756)

**A****IDENTITY**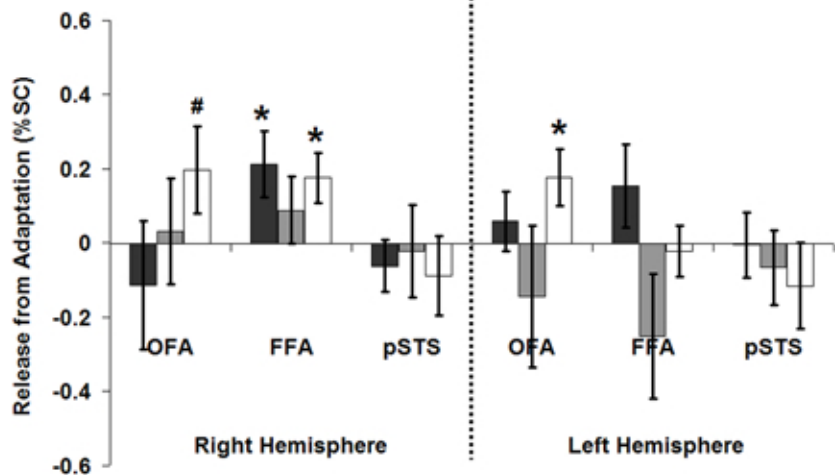**B****EXPRESSION**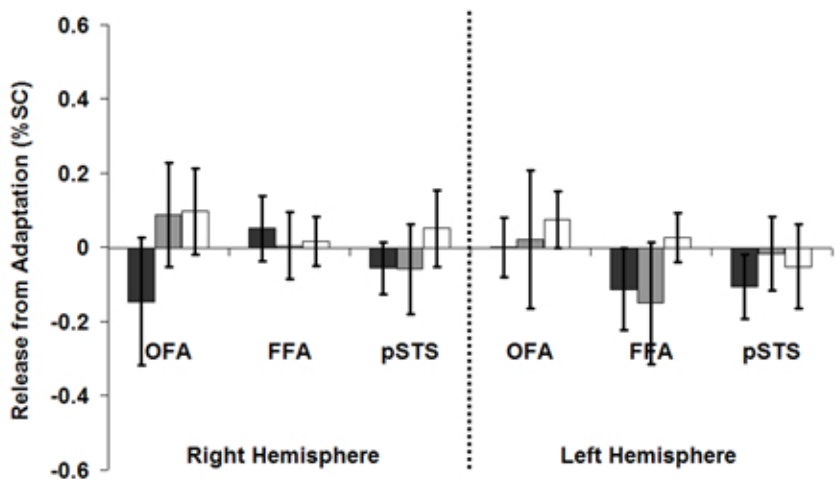

Supplement: Supplemental Figure 1 — (A) Control data for the different-identity/same-expression > same-identity/same-expression contrast. A significant release from adaptation (*) for identity changes was seen within the right FFA of C01 and C03, and within the left OFA of C03. A trend in the same direction (#) was observed in the right OFA of C03. (B) No significant release from adaptation was observed for changes in expression, following the same-identity/different-expression > same-identity/same-expression contrast. When compared to the data from the patient population we again see a release from adaptation to identity changes in the right FFA (2/3 controls) but there is no evidence of sensitivity to facial expression with this experimental design. [file Presentation1.PDF]
